# An engineered antibody fragment targeting mutant β-catenin via major histocompatibility complex I neoantigen presentation

**DOI:** 10.1074/jbc.RA119.010251

**Published:** 2019-11-05

**Authors:** Michelle S. Miller, Jacqueline Douglass, Michael S. Hwang, Andrew D. Skora, Michael Murphy, Nickolas Papadopoulos, Kenneth W. Kinzler, Bert Vogelstein, Shibin Zhou, Sandra B. Gabelli

**Affiliations:** ‡ Department of Oncology, Johns Hopkins University School of Medicine, Baltimore, Maryland 21287; § Ludwig Center, Sidney Kimmel Comprehensive Cancer Center, Johns Hopkins University School of Medicine, Baltimore, Maryland 21287; ¶ GE Healthcare Life Sciences, Marlborough, Massachusetts 01752; ‖ Howard Hughes Medical Institute, Johns Hopkins University School of Medicine, Baltimore, Maryland 21287; ** Department of Medicine, Johns Hopkins University School of Medicine, Baltimore, Maryland 21287; ‡‡ Department of Biophysics and Biophysical Chemistry, Johns Hopkins University School of Medicine, Baltimore, Maryland 21205

**Keywords:** beta-catenin, immunotherapy, major histocompatibility complex (MHC) class I, phage display, antibody engineering, catenin beta 1 (CTNNB1), human leukocyte antigen (HLA), neoantigen, MANAbody, cancer

## Abstract

Mutations in *CTNNB1*, the gene encoding β-catenin, are common in colon and liver cancers, the most frequent mutation affecting Ser-45 in β-catenin. Peptides derived from WT β-catenin have previously been shown to be presented on the cell surface as part of major histocompatibility complex (MHC) class I, suggesting an opportunity for targeting this common driver gene mutation with antibody-based therapies. Here, crystal structures of both the WT and S45F mutant peptide bound to HLA-A*03:01 at 2.20 and 2.45 Å resolutions, respectively, confirmed the accessibility of the phenylalanine residue for antibody recognition. Phage display was then used to identify single-chain variable fragment clones that selectively bind the S45F mutant peptide presented in HLA-A*03:01 and have minimal WT or other off-target binding. Following the initial characterization of five clones, we selected a single clone, E10, for further investigation. We developed a computational model of the binding of E10 to the mutant peptide–bound HLA-A3, incorporating data from affinity maturation as initial validation. In the future, our model may be used to design clones with maintained specificity and higher affinity. Such derivatives could be adapted into either cell-based (CAR-T) or protein-based (bispecific T-cell engagers) therapies to target cancer cells harboring the S45F mutation in *CTNNB1*.

## Introduction

Activation of the canonical Wnt/β-catenin pathway has been implicated in the pathogenesis of many malignancies. Signaling occurs via the binding of Wnt proteins to members of the Frizzled receptor family. This disrupts the formation of the multiprotein destruction complex involving β-catenin, adenomatous polyposis coli (APC)^4^ and AXIN, increasing the cytosolic levels of β-catenin and targeting it to the nucleus. In the nucleus, β-catenin forms a transcription activation complex with TCF/LEF transcription factors. In the absence of Wnt ligands, the formation of the multiprotein complex leads to phosphorylation of β-catenin by GSK-3β and CSK 1-α, targeting it for degradation by the proteasome (1). *CTNNB1* mutations are the second most common way of inactivating this pathway (with APC the most common) (2). The majority of *CTNNB1* mutations occur at phosphorylation sites, which prevent proteasomal targeting and lead to accumulation of β-catenin (3–6). Approximately 25% of all hepatocellular carcinomas, one of the most common cancers in the world, have mutations in *CTNNB1*. These mutations predominantly affect Ser-45 of β-catenin (7). Approximately 25% of mismatch repair–deficient colon cancers have *CTNNB1* mutations (8). Somatic mutations in *CTNNB1* are also common in desmoid type fibromatosis (DF), a rare type of soft tissue sarcoma, with up to 85% of sporadic DF harboring mutations. In particular, mutation of Ser-45 to phenylalanine (9), which makes up ∼33% of *CTNNB1* mutations in DF, results in a more aggressive phenotype, and tumors are more likely to recur after excision of the primary tumor (10).

β-Catenin has historically been considered an “undruggable” target, lacking the deep-binding sites found on enzymes and receptors, and as a result, screening efforts have primarily focused on other pathway members (11). In addition to shallow binding sites, β-catenin binds multiple effector proteins, with both activators and repressors binding to the same site (12). Nevertheless, some groups have identified small molecules (13–22) or peptides/peptidomimetics (23–27) that directly bind β-catenin and show cellular inhibition of Wnt pathway signaling. However, the direct reliance of this phenotypic effect on small-molecule binding to β-catenin has yet to be established (11). Although progress has been made, the druggability of β-catenin has yet to be shown unequivocally.

Cancer immunotherapy is a growing field that has shown unprecedented success in the clinic. For example, CD19-targeting chimeric antigen receptor T cells (CAR-Ts) and the bispecific T-cell engager (BiTE) blinatumomab have efficacy against CD19-bearing tumors, such as multiple myeloma and acute lymphoblastic leukemia (28, 29). Whereas CD19 is expressed on both normal and cancerous B-cells, normal B-cells can be depleted without adverse effects on patients. However, there are a dearth of cell surface proteins that are only expressed in cancer cells, and few of the normal tissues bearing the proteins are expendable (30). Alternatively, antibody-drug conjugates (ADCs), where a cytotoxic drug is conjugated to an antibody, are another form of antibody-based cancer treatment, such as the Food and Drug Administration–approved ado-trastuzumab emtansine, which targets Her2, a tumor-associated antigen overexpressed on a subset of breast cancers (31). Antibody-based therapies, including CAR-Ts, BiTEs, and ADCs, provide an important alternative for “difficult-to-drug” targets, such as β-catenin, but are limited by the cytosolic or nuclear localization of most tumor-associated or tumor-specific targets.

To overcome the inherent challenge of targeting intracellular proteins with antibody or antibody fragment–based therapies, one can instead target peptides derived from these proteins that are presented on the cancer cell surface as part of a complex with major histocompatibility complex class I (MHC-I) proteins. These complexes include peptides derived from tumor-associated proteins and tumor-specific mutant proteins, the latter class of peptides referred to as mutation-associated neoantigens (MANAs). Such MHC-I complexes can be targeted via T-cell receptors, either directly through tumor-infiltrating lymphocytes (32) and transgenic TCRs (33, 34) or indirectly through anti-CTLA4/anti-PD1 checkpoint blockade (35). TCR-mimic antibodies can be developed that specifically recognize the target MHC-I. A TCR-mimic antibody targeting a Wilms tumor 1 (WT1) peptide in the context of HLA-A2 has demonstrated efficacy in preclinical models (36); however, the ability to specifically target MANAs is critical to make these strategies applicable to a wide range of cancer patients.

MHC class I complexes are formed between human leukocyte antigen (HLA) proteins, β_2_-microglobulin, and a peptide, typically 8–10 amino acids in length, derived from an intracellular protein. The HLA family of proteins are anchored in the plasma membrane by a single transmembrane helix. The extracellular portion is made up of a “stalk” formed by a single Ig-like domain, and the peptide-binding domain. The peptide-binding domain is formed between two α-helices, with an eight-stranded β-sheet forming the base. β_2_-Microglobulin is a single domain protein with a typical Ig-like fold and binds alongside the “stalk” to further support the HLA peptide-binding domain. Peptide binding is mediated by two anchoring residues, the second (P2) and C-terminal amino acids of the peptide, which bind in pockets “B” and “F”, respectively, and anchor the rest of the peptide (37). Different HLA allotypes bind peptides with different characteristics. For example, HLA-A3 binds peptides with a small, aliphatic residue at P2 and a basic residue at the C terminus, whereas HLA-A24 binds peptides with aromatic or aliphatic residues at P2 and aromatic, aliphatic, or hydrophobic residues at the C terminus (37).

Previously, we have reported the use of phage display to identify scFvs (single-chain variable fragments) that specifically recognize peptides derived from common oncogene mutations in common HLA alleles (KRAS G12V in HLA-A2 and EGFR L858R in HLA-A3) and converted these scFvs into full-length antibodies called “MANAbodies” (for mutation-associated neoantigen antibodies) (38). Herein, we report the application of this approach to a new target, a β-catenin S45F mutant neoantigen presented by HLA-A3, which represents an important alternative to traditional small molecule–based therapies. We identify scFvs specifically targeting the β-catenin neoantigen that could serve as building blocks for the development of antibody-based therapies for cancers harboring *CTNNB1* mutations.

## Results

### Target peptide selection: S45F is exposed upon β-catenin_41–49_ peptide binding to HLA-A*03:01

We sought to identify a target mutant β-catenin peptide that would have a high likelihood of being endogenously processed and presented as part of the MHC class I complex. Using the COSMIC database and NetMHCv4.0, we identified peptides containing common driver gene mutations from *CTNNB1* that were predicted to bind to common HLA alleles. The WT versions of these peptides were searched in the Immune Epitope Database for those with evidence of presentation in the predicted HLA allele. The WT β-catenin peptide TTAPSLSGK (amino acids 41–49; hereafter “WT peptide”) has been previously shown to be presented on human B cells using MS, likely in HLA-A3 (39, 40). The analogous mutant S45F β-catenin peptide, TTAP**F**LSGK (amino acids 41–49, hereafter “mutant peptide”), is predicted to bind to HLA-A3 with high affinity (28 nm, NetMHCv4.0 (41, 42)). HLA-A*03:01 has an allele frequency of ∼2.5% in Asian Americans, 8% in Hispanic and African Americans, and 14% in Caucasian Americans (Allele Frequency Net Database) (43).

**Figure 1.**
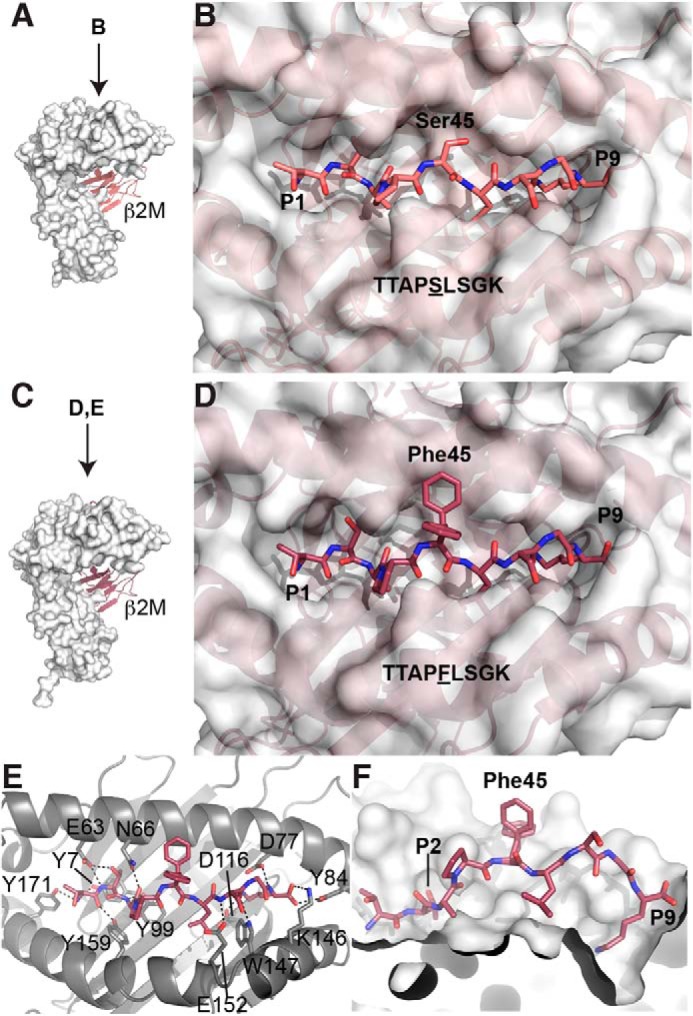
WT and S45F mutant β-catenin peptides bind HLA-A*03:01 with S45F exposed. *A*, overall structure of HLA-A3 bound to the WT β-catenin peptide (residues 41–49) (PDB entry 6O9B). The HLA-A3 complex is formed as a heterodimer between the heavy chain, represented as a *white surface*, and β_2_-microglobulin (β*2m*), represented as a *cartoon*. In this orientation, the peptide (not visible) binds in a cleft at the top of the heavy chain from *left* to *right. B*, *bird's eye view* of the WT peptide bound to HLA-A3. The peptide binds in a cleft between the α1 and α2 helices of HLA-A3. The amino acid residues are defined as P1–P9; the residue of interest, β-catenin Ser-45, is in the P5 position and is accessible for binding by an antibody. *C*, the overall structure of HLA-A3 bound to the S45F mutant β-catenin peptide (residues 41–49) (PDB entry 6O9C), in the same orientation as the WT structure in *A*. The heavy chain is represented by a *white surface*, and β_2_-microglobulin is shown in a *cartoon. D*, *bird's eye view* of the mutant peptide bound to HLA-A3. The surface of HLA-A3 is shown. *E*, detailed interactions of the mutant peptide with HLA-A3. The peptide and the relevant side chains of interacting residues are represented as *sticks*. Hydrogen bonds are shown as *dashed lines*. The peptide is anchored by multiple hydrogen bonds on either end. Two conformations of the mutant β-catenin residue of interest, Phe-45, are shown. *F*, *cut-away view* of the peptide-binding cleft. The α2 helix has been removed so that the inner surface of the binding cleft is visible. The anchoring pockets for the residues at P2 and P9 are marked. The two conformations of Phe-45 are shown.

Within a given peptide displayed in MHC class I, individual amino acid residues can be partially or completely exposed or can be buried and inaccessible to antibody binding. To identify scFvs specific for the mutant peptide, it was important for residue 45 to be exposed and available for binding. To confirm the binding of these peptides to HLA-A3 and the suitability for antibody generation, we determined the structures of both the WT and mutant β-catenin peptide-bound HLA-A3 (WT/mutant pHLA-A3). Inclusion bodies of HLA-A3 heavy chain and β_2_-microglobulin were refolded in the presence of either WT or mutant peptide and purified via size-exclusion chromatography. The structure of the WT pHLA-A3 was determined to 2.2 Å, and that of the mutant pHLA-A3 was determined to 2.45 Å (Table 1). Both WT and mutant pHLA-A3 exhibit the canonical fold consistent with previously published structures of HLA-A3, with an RMSD of 0.483 Å between them (44, 45) (Fig. 1*A*). The peptides bind in the cleft between the α1 and α2 helices, differing in only a single amino acid residue, located at the P5 position. The peptides are anchored in the HLA-A3 structure by binding of threonine at P2 and lysine at P9. In the WT pHLA-A3 structure, the serine at P5 orients laterally, making it accessible to antibody binding (Fig. 1*B*). In the mutant pHLA-A3 structure (Fig. 1*C*), all peptide residues except for P5 bind in identical conformations to the WT pHLA-A3 (Fig. 1, *D–F*). Interestingly, electron density for two distinct conformations of the phenylalanine residue at P5 were observed (Fig. S1). In one conformation, the phenylalanine side chain sticks out of the binding cleft, exposed for antibody binding, whereas the other conformation orients the side chain laterally, similar to the serine side chain in the WT structure. The side chain of the phenylalanine residue is not anchored by any interactions with residues from the HLA-A3 protein, making it flexible and able to adapt to binding by an antibody. Both conformations should be accessible to binding, and a single final bound conformation may be stabilized by antibody binding. Thus, the mutant S45F β-catenin peptide (amino acids 41–49) was an ideal candidate for MANAbody generation.

**Table 1:** **Data collection and refinement statistics** Values for the highest-resolution shell are shown in parentheses.

	WT β-catenin_41–49_-HLA-A*03:01 (PDB entry 6O9B)	S45F β-catenin_41–49_-HLA-A*03:01 (PDB entry 6O9C)
**Data collection**		
Diffraction source	NSLS-II X17-ID-2	NSLS-II X17-ID-1
Wavelength (Å)	0.979000	0.918394
Temperature (K)	100	100
Detector	Dectris EIGER X 16M	Dectris EIGER X 9M
Rotation range per image (degrees)	0.1	0.2
Total rotation range (degrees)	96	160
Space group	p622	p622
*a*, *b*, *c* (Å)	152.77, 152.77, 84.83	155.12, 155.12, 85.32
α, β, γ (degrees)	90.00, 90.00, 120.00	90.00, 90.00, 120.00
Resolution range (Å)	28.89–2.20 (2.26–2.20)	44.78–2.45 (2.52–2.45)
Total no. of observations	313,372	409,173
No. of unique observations	29,999	22,620
Completeness (%)	99.2 (91.2)	99.9 (99.7)
Redundancy	10.4 (9.8)	18.1 (18.8)
〈*I*/σ(*I*)〉	9.7 (2.1)	13.9 (2.1)
*R*_merge_	0.157 (1.03)	0.173 (1.46)
CC_½_	0.996 (0.704)	0.998 (0.767)
**Refinement**		
Resolution range (Å)	28.89–2.20 (2.25–2.20)	44.78–2.45 (2.52–2.45)
No. of reflections, working set	28,546	21,494
No. of reflections, test set	1,452	1,132
*R*_work_/*R*_free_	0.182/0.227 (0.231/0.226)	0.214/0.259 (0.321/0.361)
No. of non-H atoms		
Protein	3,175	3,177
Ligand/ion	133	88
Water	166	43
RMSD		
Bonds (Å)	0.016	0.010
Angles (degrees)	1.93	1.62
Average *B* factors (Å^2^)		
Protein	39.8	50.7
Ligand/ion	63.1	89.8
Water	43.0	42.1
Ramachandran (%)		
Favorable	97.7	96.9
Allowed	2.3	2.6
Disallowed	0	0.5

### Identification of S45F mutant β-catenin_41–49_ S45F-HLA-A*03:01–specific phage clones

We sought to identify scFvs that specifically bound to β-catenin_41–49_ S45F-HLA-A*03:01 via phage display. The WT and mutant peptides, along with other control HLA-A3–binding peptides were synthesized and folded into HLA-A3/β_2_-microglobulin complexes. pHLA-A3 complexes were biotinylated for use in further assays. We used two scFv-phage display libraries for panning, both based on the humanized 4D5 framework (46), chosen for its successful use in other previously published phage display libraries (47, 48). In our first library (38), variability was introduced in particular codons of four CDRs (L3, H1, H2, and H3) (48) using degenerate oligonucleotide mixtures. A subsequent library was designed and built that varied five CDRs (L2, L3, H1, H2, and H3) using trinucleotide mutagenesis (TRIM) technology to achieve greater amino acid diversity in specified positions. In both libraries, the specific amino acid diversity found at particular CDR positions was either chosen based on its presence in the human antibody repertoire or because it had been previously demonstrated to play a significant role in antigen binding (49).

Panning was performed over six rounds of selection, which was divided into three distinct amplification phases: an enrichment phase (round 1), a competitive phase (rounds 2–4), and a final selection phase (rounds 5 and 6) (38). We divided the panning into these three phases to slowly enrich for mutant pHLA-A3–specific phage. In each subsequent round, decreased amounts of precipitated phage from the previous round were used as input for the subsequent round.

**Figure 2.**
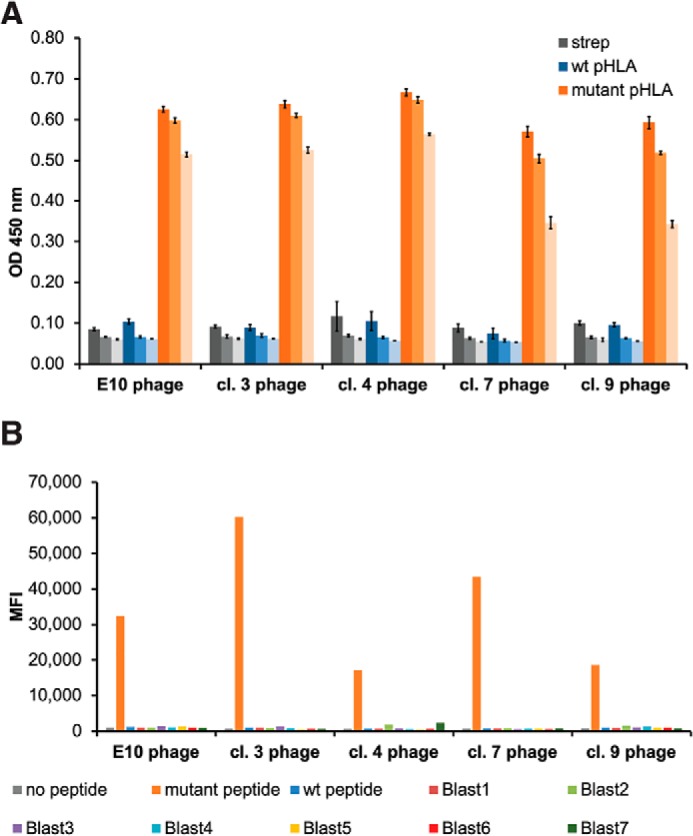
Identification of S45F mutant β-catenin_41–49_-HLA-A*03:01–specific phage clones. *A*, five phage clones bind specifically to the β-catenin mutant pHLA-A3 by ELISA. pHLA-A3 complexes or streptavidin bound to plates were incubated with dilutions of the various phage clones (1:500, 1:2,500, and 1:12,500 from darkest to lightest in each color series), followed by detection with an anti-M13 antibody. Data are represented as mean ± S.D. (*error bars*). *B*, five phage clones bind specifically to mutant pHLA-A3 on the cell surface. T2A3 cells were peptide-pulsed with β_2_-microglobulin in the presence or absence of the specified peptide and then stained with the various phage clones and analyzed by flow cytometry. No significant binding was detected to any of the seven Blast peptides.

Following panning, we used monoclonal ELISAs to evaluate the binding of individual phage clones to the mutant and WT pHLA-A3 complexes. Monoclonal ELISAs were performed on phage from rounds 4, 5, and 6 to capture both the most enriched phage clones (phage from later rounds) and any phage clones that may have been eliminated by bottlenecking (phage from earlier rounds). Phage clones with high mutant pHLA-A3 binding and low WT pHLA-A3 binding were sequenced, and unique phage clones were amplified for further characterization on ELISAs. Using this method, we identified five phage clones specific for the mutant pHLA-A3 (Fig. 2*A*). One of these clones (“E10”) was derived from the first phage library, and four (clones 3, 4, 7, and 9) were derived from the second library (see Fig. S2 for sequences). ELISAs were performed with serial dilution of each phage clone to assess binding to the mutant pHLA-A3, WT pHLA-A3, or streptavidin as a control (Fig. 2*A*).

We sought to determine whether these phage clones could specifically bind the mutant β-catenin pHLA-A3 on the cell surface using the cell line T2A3. T2A3 cells are a stably transfected HLA-A3 variant of the TAP-deficient T2 cell line that expresses only low levels of HLA-A3 on its surface without the addition of exogenous HLA-binding peptide and β_2_-microglobulin (50, 51). T2A3 cells were pulsed with the mutant or WT peptide in the presence of β_2_-microglobulin followed by staining with phage and anti-M13 secondary antibodies. Although similar levels of pHLA-A3 were present on the cell surface after pulsing with the mutant or WT peptide (Fig. S3), all five clones exhibited specific staining for only the mutant peptide-pulsed T2A3 cells (Fig. 2*B*).

To further assess the specificity of these five phage clones for peptides derived from proteins that might be presented on normal human cells, we identified so-called “BLAST” peptides by comparing the mutant peptide with peptides from the normal human proteome. These peptides were identified using the human refseq database and BLASTp, followed by netMHCv4.0 to predict their HLA-A3–binding capacity. We thereby identified seven peptides with similarity to the mutant peptide (Table S1) and found that they each stabilized HLA-A3 on the surface of T2A3 cells to varying degrees (Fig. S3). Remarkably, none of these seven peptides exhibited cross-reactivity with any of the phage clones (Fig. 2*B*).

### Evaluation of scFv binding to S45F mutant and WT β-catenin_41–49_-HLA-A*03:01

**Figure 3.**
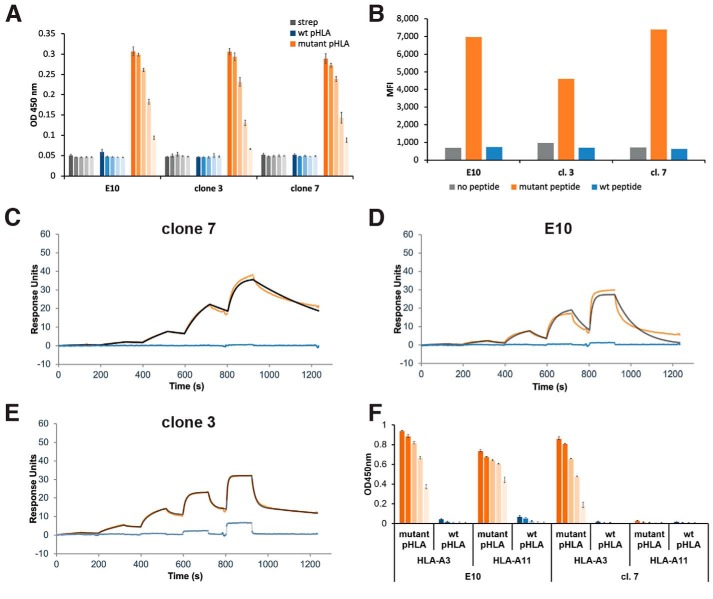
Evaluation of scFv binding to S45F mutant and WT β-catenin_41–49_-HLA-A*03:01. *A*, analysis of recombinant scFv binding to mutant pHLA by ELISA. pHLA-A3 complexes or streptavidin bound to plates were incubated with decreasing concentration of scFvs (200, 40, 8, 1.6, and 0.64 nm from darkest to lightest in each color series). scFv binding was detected with protein L followed by an anti-protein L antibody. Data are represented as the average of three technical replicates ± S.D. (*error bars*). *B*, analysis of recombinant scFv binding to peptide-pulsed cells by flow cytometry. T2A3 cells were peptide-pulsed with β_2_-microglobulin and the specified peptide, stained with the various scFvs, and analyzed by flow cytometry. *C–E*, scFv binding to mutant and WT pHLA-A3 was evaluated by single-cycle kinetics using SPR. The scFv was loaded at increasing concentrations from 6.25 to 25, 100, and 400 nm for cl. 7 and cl. 3 and 15.6, 62.5, 250, and 1000 nm for E10. Blank- and reference-subtracted binding responses are shown in *orange* for the mutant pHLA-A3 and *blue* for the WT pHLA-A3; the *fitted curve* is shown in *black* or *gray. C*, cl. 7 displays one-to-one binding to the mutant pHLA-A3 and minimal binding to the WT pHLA-A3. *D*, E10 displays one-to-one binding to the mutant pHLA-A3 and minimal binding to the WT pHLA-A3. *E*, cl. 3 displays heterogeneous ligand binding to the mutant pHLA-A3 and displays some off-target binding to the WT pHLA-A3 at 100 and 400 nm. *F*, evaluation of cl. 7 and E10 binding to the mutant and WT peptides in complex with HLA-A*03:01 and HLA-A*11:01 by ELISA. Data are represented as the average of three technical replicates ± S.D.

We selected the phage clones that showed the strongest response to peptide-pulsed T2A3 cells for further analysis: E10, cl. 7, and cl. 3. Each clone was expressed in either bacteria or HEK293F cells and purified by affinity and size-exclusion chromatography. Each scFv showed a dilutional binding response to the mutant pHLA-A3 by ELISA, with no binding to WT pHLA-A3 at concentrations up to 200 nm (Fig. 3*A*). Similar selectivity was observed with peptide-pulsed cells analyzed by flow cytometry (Fig. 3*B*).

To quantitatively evaluate the kinetics and affinity of each of these scFvs for both the WT and mutant pHLA-A3, we used surface plasmon resonance (SPR). Biotinylated WT and mutant pHLA-A3 complexes were separately loaded onto individual flow cells of a streptavidin sensor chip. The binding of the scFvs to both WT and mutant pHLA-A3 was then assessed via single-cycle kinetics (Table 1 and Fig. 3 (*C–E*)). Cl. 7 displayed one-to-one binding kinetics, with the *K_D_* to the mutant pHLA-A3 determined to be 28.5 nm (Table 2 and Fig. 3*C*). No significant response to the WT pHLA-A3 was observed (Fig. 3*C*). E10 also displayed one-to-one binding kinetics (*K_D_* = 150 nm) and no significant WT pHLA-A3 binding (Table 2 and Fig. 3*D*). In contrast, the shape of the binding response from cl. 3 suggested a heterogeneous ligand-binding model to the mutant pHLA-A3 (Fig. 3*E*). The high-affinity binding site had a *K_D_* of 2 nm and, due to its specificity to the mutant pHLA-A3, likely incorporated the phenylalanine residue of the mutant peptide. The lower-affinity binding site was more likely to be nonspecific, binding to parts of the peptide that were in common with the WT or to only the HLA. In line with this result, clone 3 showed some low-affinity WT pHLA-A3 binding (*K_D_* = 1,600 nm) (Table 2 and Fig. 3*E*). Clone 3 was thus excluded from further evaluation due to its cross-reactivity with the WT pHLA.

**Table 2:** **Kinetics and affinity of scFv binding to mutant and WT pHLA-A3 determined by SPR** ND, not determined; NA, not applicable.

	S45F β-catenin_41–49_-HLA-A*03:01				WT β-catenin_41–49_-HLA-A*03:01			
	*k*_on_	*k*_off_	*K_D_*	*t*_½_	*k*_on_	*k*_off_	*K_D_*	*t*_½_
	*m*^−*1*^*s*^−*1*^	*s*^−*1*^	*nm*	*s*	*m*^−*1*^*s*^−*1*^	*s*^−*1*^	*nm*	*s*
Clone 7	7.24 × 10^4^	2.06 × 10^−3^	28.5	336	ND	ND	>400	ND
E10	6.56 × 10^4^	9.86 × 10^−3^	150	70	ND	ND	>1000	ND
Clone 3 binding site	4.27 × 10^5^	8.40 × 10^−4^	1.97	825	NA	NA	NA	NA
Clone 3 binding site	2.61 × 10^5^	6.34 × 10^−2^	243	11	2.64 × 10^5^	4.39 × 10^−1^	1,600	2

To evaluate the HLA specificity of our clones, we evaluated the binding of cl. 7 and E10 to both mutant and WT peptide–bound HLA-A*03:01 and HLA-A*11:01 complexes. Both HLA-A*03:01 and HLA-A*11:01 are part of the A3 superfamily and are expected to bind similar peptides. The two allotypes differ by only seven amino acids, four of which are in the peptide-binding domain. Interestingly, E10 binds the mutant peptide in both HLA-A*03:01 and HLA-A*11:01, whereas cl. 7 only binds the peptide in the HLA-A*03:01 complex (Fig. 3*F*).

### Characterization of scFv clones via complement-dependent cytotoxicity assays

**Figure 4.**
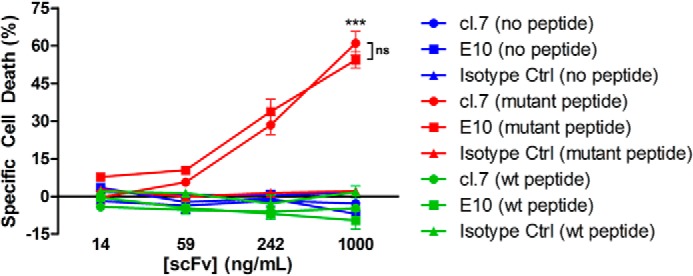
**ScFv-mediated, complement-dependent cell killing.** T2A3 cells were peptide-pulsed with β_2_-microglobulin in the presence or absence of the specified peptide: no peptide (*blue*), the S45F mutant peptide (TTAPFLSGK) (*red*), or the WT peptide (TTAPSLSGK) (*green*). Cells were incubated with 10% rabbit serum and the cl. 7, E10, or isotype control scFvs preconjugated to anti-V5 antibody. CellTiter-Glo was used to assess the viability of cells. ***, *p* < 0.0001, comparing either cl.7 or E10 with isotype control at the highest antibody concentration with mutant peptide-pulsed cells; *ns*, not significant (*p* = 0.1130). *Error bars*, S.D.

The scFvs have potential therapeutic utility as building blocks for a variety of antibody-based therapies: BiTEs, CAR-Ts, or ADCs. To show the capability of these scFvs to mediate targeted cell killing, we performed a complement-dependent cytotoxicity (CDC) assay. We first pulsed T2A3 cells with mutant or WT peptide in the presence of β_2_-microglobulin. Then we performed a CDC assay using the V5-tagged E10 or cl. 7 scFvs preconjugated to an anti-V5 antibody. The W6/32 antibody, which recognizes any correctly folded class I HLA protein (52), was used as a positive control. Both scFvs E10 and cl. 7 resulted in selective mutant peptide-pulsed cell death in a dilutional manner, unlike the isotype control scFv (Fig. 4). In contrast, T2A3 cells pulsed with the WT peptide plus β_2_-microglobulin or β_2_-microglobulin alone exhibited marginal cell death (*p* < 0.0001).

### Affinity maturation and computational modeling

Despite their differences in affinity as measured by SPR, cl. 7 and E10 showed no significant differences in their ability to kill cells via the CDC assay. To prioritize a single clone for further development, we investigated the aggregation propensity of each clone. A recent publication has reported the higher aggregation potential of antibodies discovered via phage display (53) and the associated negative correlation with clinical success. It is therefore important to prioritize antibodies based not only on affinity, but also those with low aggregation potential. Using an aggregation propensity algorithm, we calculated an aggregation score, AggScore, for both cl. 7 and E10 (54). Cl. 7 had an AggScore of 142.8, whereas E10 had a score of 67.3. In addition to its low predicted aggregation potential, E10 has wider specificity, binding to the mutant peptide in both HLA-A*03:01 and HLA-A*11:01, which would make it potentially useful for a wider patient pool. We thus selected E10 for further development.

**Figure 5.**
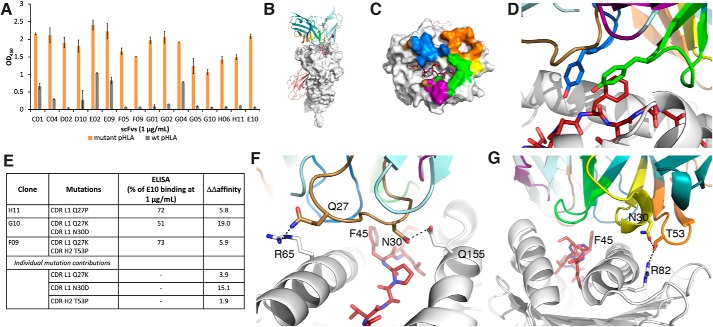
Development of a model of E10 binding to mutant β-catenin_41–49_-HLA-A*03:01. *A*, ELISA of affinity-matured clones. *B*, model of E10 binding to S45F β-catenin_41–49_-HLA-A*03:01. HLA-A3 is shown as a *white surface*. β_2_-Microglobulin is shown as a *salmon cartoon*. The light chain of the scFvs is *colored* in *light teal*, and the heavy chain in *dark teal*, with the CDR loops *colored* as follows: L1 (*brown*), L2 (*purple*), L3 (*blue*), H1 (*yellow*), H2 (*orange*), and H3 (*green*). The mutant peptide is shown as *red sticks* throughout. *C*, E10 buries 2,113 Å^2^ of surface area. The contact surface on HLA-A3 for each of the CDRs has been *colored* accordingly. *D*, Phe-45 is recognized by tyrosine residues on CDR L3 and H3. *E*, *table* of single and double mutants from affinity maturation studies. Experimental differences in binding and calculated ΔΔaffinity values are compared. *F*, CDR L1 residues Gln-27 and Asn-30 make hydrogen-bond interactions with HLA-A3 residues Arg-65 and Gln-155, respectively. These interactions would be disrupted upon mutation to proline or lysine. *G*, CDR H2 residue Thr-53 forms a hydrogen bond with Arg-82 on the HLA, along with a stabilizing interaction with Asn-30 on loop H1. These interactions would be disrupted upon mutation to proline. *Error bars*, S.D.

We proceeded to attempt to affinity-mature E10. Briefly, a DNA library with NNN degenerate codons skewed toward the parental codon in all six CDRs was synthesized and cloned into a phagemid vector. The resulting phage library was subjected to three rounds of panning. Each round of panning consisted of negative selection against WT pHLA-A3 and positive selection against mutant pHLA-A3. The evaluated clones had 1–5 mutations concentrated in the CDRs. Unfortunately, the only clones that showed an increase in mutant pHLA-A3 binding, as determined by ELISA, also showed increased binding to WT pHLA-A3 and thus a loss of selectivity (Fig. 5*A*).

Data from the affinity maturation clones with a single or double mutation were then used to develop a model of the binding of E10 to the mutant pHLA-A3. This model may be used to provide a structural rationale for future clone development. We predicted the structure of E10 and then docked it to our mutant pHLA-A3 crystal structure. Poses that failed to interact with the key phenylalanine residue were excluded. Four poses that displayed good recognition of the mutant phenylalanine residue were then energy-minimized. The top pose was selected based on its ability to explain experimental data from the single and double mutants and the dual HLA-A*03:01 and HLA-A*11:01 binding (Fig. 5*B*).

According to the model, binding of the scFv to the pHLA-A3 buries 2,113 Å^2^ of surface area, with a 61% apolar contribution (Fig. 5*C*). The phenylalanine residue of interest is recognized on two sides by tyrosines from loops L3 and H3 (Fig. 5*D*). No contact is made with the nonconserved residues between HLA-A*03:01 and HLA-A*11:01.

The clone H11 harbors a single point mutation from a glutamine to a proline on loop L1 and results in a reduction in binding by ELISA of ∼30% at 1 μg/ml scFv (Fig. 5*E*). In the model, Gln-27 forms a charge-assisted hydrogen bond with HLA-A3 Arg-65 (Fig. 5*F*). Mutation of the glutamine to a proline would result in loss of this interaction, in addition to potentially altering the conformation of the loop. *In silico* prediction of ΔΔaffinity of the Q27P mutation gives a value of 5.8, predicting a significant loss of affinity (Fig. 5*E*).

Double mutant clones G10 and F9 both have a mutation of the same glutamine to lysine (Q27K) in CDR L1 (Fig. 5*E*). This single mutation has a predicted ΔΔaffinity of 3.9. The second mutation in G10 is at position 30 in the same L1 loop, N30D. The combination of the two mutations results in a 50% loss of binding at 1 μg/ml scFv concentration. Computing the ΔΔaffinity of these two mutations based on our model gives a value of 19.0, with the largest contribution from N30D (ΔΔaffinity of 15.1). In our model, Asn-30 interacts with Gln-155 on HLA-A3 (Fig. 5*F*).

The second mutation in F9 is in the loop H2, at T53P, and the combination of this mutation with Q27K results in a 30% loss of binding at 1 μg/ml scFv concentration. F9 has a total predicted ΔΔaffinity of 5.9, with a slightly larger contribution from Q27K (ΔΔaffinity = 3.9) compared with T53P (ΔΔaffinity = 1.9) (Fig. 5*E*). In our model, Thr-53 forms a hydrogen bond with Arg-82 on HLA-A3, which would be lost upon mutation to proline (Fig. 5*G*). Furthermore, Thr-53 forms a hydrogen bond with Asn-30 in CDR loop H2, potentially contributing to the stability and conformation of the scFv. Future work will involve using our validated model to design new mutants to bind the mutant pHLA-A3 more tightly without increasing WT pHLA-A3 binding.

## Discussion

We have presented a novel method for potentially targeting tumors harboring mutations in *CTNNB1*. In particular, we successfully identified two scFv clones that selectively target a β-catenin mutant neoantigen presented in HLA-A3, with minimal WT binding. These scFv clones have the potential to be used as building blocks for the development of various modes of immunotherapies, such as full-length antibodies, ADCs, CAR-Ts, and BiTEs.

In this situation, affinity maturation was ultimately unsuccessful at identifying derivatives of E10 with increased mutant pHLA-A3 affinity and maintained selectivity over WT pHLA-A3. However, we were able to use the data to develop a computational model of E10 binding to the mutant pHLA-A3 that is supported by experimental evidence. This model will be used to design new E10 derivatives to increase the affinity while maintaining selectivity. The model will be continually refined with the input of further experimental data. In parallel, we will pursue affinity maturation studies of cl. 7, focusing also on reducing its aggregation potential.

In conclusion, by exploiting the MHC presentation of β-catenin peptides, we have achieved the first step of an approach to selectively target this important mutated driver gene product. The exquisite selectivity achievable with antibodies provides the added benefit of distinguishing between WT and mutant proteins—the foundation for developing effective treatments with minimal adverse effects to patients. Further studies will investigate whether various recombinant derivatives of the scFvs derived from E10 can recognize the low endogenous levels of surface HLA-A3-mutant β-catenin peptides and destroy cancer cells that harbor them.

## Experimental procedures

### Cell lines

T2A3 cells (a kind gift from Eric Lutz and Elizabeth Jaffee, Johns Hopkins University) were cultured in RPMI 1640 (ATCC, Manassas, VA) with 10% HyClone fetal bovine serum (GE Healthcare), 1% penicillin-streptomycin (Life Technologies, Inc.), 500 μg/ml Geneticin (Life Technologies), and 1× non-essential amino acids (Life Technologies) at 37 °C under 5% CO_2_.

### Peptides

All peptides were synthesized at a purity of >90% by Peptide 2.0 (Chantilly, VA). Peptides were resuspended in DMSO at 10 mg/ml and stored at −80 °C. Peptides used included the following: WT β-catenin_41–49_ (Uniprot P35222) peptide (TTAPSLSGK), S45F mutant β-catenin_41–49_ peptide (TTAPFLSGK), peptides identified via BLAST with similarity to the mutant β-catenin peptide blast 1 (QLLDFLSGK), blast 2 (SLNPKFLSGK), blast 3 (IIYNFLSGK), blast 4 (RTVTFLSGK), blast 5 (TAFDPFLGGK), blast 6 (RIIPFLPGK), blast 7 (IQNPFLSSK), and negative control HLA-A3–binding peptides from WT KRAS (VVGAGGVGK) and WT EGFR (KITDFGLAK).

### pHLA-A3 production

pHLA-A3 complexes used for phage display and ELISAs were prepared by refolding recombinant HLA with peptide and β_2_-microglobulin, purified by size exclusion, and biotinylated (Fred Hutchinson Immune Monitoring Laboratory (Seattle, WA) and Baylor MHC Tetramer Core (Houston, TX)). pHLA-A3 complexes were confirmed to be folded prior to selection by performing an ELISA using W6/32 antibody (BioLegend, San Diego, CA), which recognizes only folded HLA. pHLA-A3 complexes used for crystallography and SPR experiments were prepared as follows. pHLA-A3 complexes used for crystallography were expressed, refolded, and purified in house. Plasmids for HLA-A3 and β_2_-microglobulin were received from the NIH Tetramer Facility (Atlanta, GA) and separately transformed into BL21(DE3) cells and expressed in inclusion bodies using autoinduction medium as described previously (57–59). The refolding was performed as described previously (58, 59). Briefly, HLA-A3 and β_2_-microglobulin inclusion bodies were combined in refolding buffer containing 100 mm Tris, pH 8.3, 400 mm l-arginine, 2 mm EDTA, 5 mm reduced GSH, 0.5 mm oxidized GSH, 2 mm phenylmethylsulfonyl fluoride, and 30 mg/liter of either the WT (amino acids 41–49, TTAPSLSGK) or mutant (amino acids 41–49, TTAPFLSGK) peptide first dissolved to 30 mg/ml in DMSO and added to the refolding buffer. The resultant mixture was stirred at 4 °C for 2 days, with two further additions of HLA-A3 inclusion bodies on day 2, concentrated to 10 ml and purified by size-exclusion chromatography on a Superdex 75 column (GE Healthcare). Purified pHLA-A3 was concentrated to 12–13 mg/ml and stored at −80 °C until use.

For SPR experiments, 10 nmol of purified pHLA-A3 at a concentration of 40 μm in 10 mm Tris, pH 8, was biotinylated with 2.5 μg of *Escherichia coli* BirA ligase (Avidity, Aurora, CO) for 1 h at 30 °C in a buffer containing 50 mm Bicine, pH 8.3, 10 mm ATP, 10 mm magnesium acetate, and 50 μm d-biotin. Excess biotin was removed by extensive dialysis.

### Crystallization, data collection, and structure determination

Crystals of the WT β-catenin_41–49_-pHLA-A*03:01 were prepared using hanging-drop vapor diffusion with a reservoir solution of 0.1 m MES/imidazole, pH 6.5, 0.03 m diethylene glycol, 0.03 m triethylene glycol, 0.03 m tetraethylene glycol, 0.03 m pentaethylene glycol, 20% (w/v) PEG 550 MME, 10% (w/v) PEG 20,000. Crystals of the S45F mutant β-catenin_41–49_-pHLA-A*03:01 were prepared using hanging-drop vapor diffusion and a reservoir solution of 0.1 m MES, pH 6.5, 0.2 m ammonium sulfate, 30% PEG 5000 MME. Data were collected at National Synchrotron Light Source-II at beamlines 17-ID-1 (mutant pHLA-A3) on a DECTRIS Eiger X 9M detector and 17-ID-2 (WT pHLA-A3) on a DECTRIS Eiger X 16M detector. Data sets were indexed, integrated, and scaled using XDS (60). The structure for the WT pHLA-A3 was determined by molecular replacement with MOLREP (61) using PDB entry 2XPG (45) as the search model. The data set was refined to a final resolution of 2.2 Å using iterative rounds of refinement with REFMAC5 (62, 63) and manual rebuilding in Coot (64). The structure for the mutant pHLA-A3 was determined by molecular replacement with MOLREP (61) using the WT pHLA-A3 structure as the search model. The data set was refined to a final resolution of 2.45 Å using iterative rounds of refinement with REFMAC5 (62, 63) and manual rebuilding in Coot (64). Structures were validated using Coot and the PDB deposition tools. Figures were rendered in PyMOL (version 2.2.3, Schrödinger, LLC, New York).

### Accession numbers

The final coordinates of WT β-catenin_41–49_-HLA-A*03:01 and S45F-β-catenin_41–49_-HLA-A*03:01 have been deposited in the PDB with accession codes 6O9B and 6O9C, respectively.

### Selection of phages

scFv-bearing phage specific to the S45F mutant β-catenin_41–49_-HLA-A*03:01 were identified following methods published previously (38). We used two phage display libraries based on the humanized 4D5 framework. The first library varies CDRs L3, H1, H2, and H3 and has been described previously (38). The second library varies CDR L2 in addition to the other four CDRs and uses TRIM technology to generate a greater degree of diversity. These two phage display libraries were subjected to panning to identify phage clones. A panning schema involving an enrichment phase, competitive phase, and final selection phase was followed. Biotinylated pHLA-A3 complexes were conjugated to M-280 streptavidin magnetic Dynabeads (Life Technologies) or to streptavidin-agarose (Novagen, Millipore, Darmstadt, Germany). The biotinylated pHLA-A3 were incubated with either 25 μl of Dynabeads beads or 100 μl of streptavidin agarose per 1 μg of pHLA-A3 in blocking buffer (PBS, 0.5% BSA, 0.1% sodium azide) for 1 h at room temperature (RT). After the initial incubation, the complexes were washed and resuspended in 100 μl of blocking buffer.

The phage display libraries, stored at −20 °C in 15% glycerol, were regrown within a week of starting the panning process. A colony of phage-competent SS320 cells (Lucigen, Middleton, WI) were inoculated in a 37 °C overnight culture of 2xYT medium (Sigma–Aldrich) supplemented with tetracycline (20 μg/ml) and the next day were grown to 2 liters of mid-log phase (*A*_600_ of 0.3–0.5) bacteria. Bacteria were infected with the phage library at an MOI of 0.5 and M13K07 Helper phage (New England Biolabs (Ipswich, MA) or Antibody Design Labs (San Diego, CA)) at an MOI of 4 along with the addition of 2% (w/v) glucose (Sigma–Aldrich) and allowed to shake for 1 h at 37 °C. The culture was centrifuged, and the cells were resuspended in 2xYT medium with carbenicillin (100 μg/ml) and kanamycin (50 μg/ml) and grown overnight at 30 °C for phage production. The following morning, the bacterial culture was aliquoted into 50-ml Falcon tubes and pelleted twice at high speed to obtain clarified supernatant. The phage-laden supernatant was precipitated on ice for 40 min with a 20% PEG 8000, 2.5 m NaCl solution at a 4:1 ratio of PEG/NaCl to supernatant. After precipitation, phage was centrifuged at 12,000 × *g* for 40 min and resuspended in 1 ml of 1× TBS with 2 mm EDTA, 0.1% sodium azide, and 1× Complete Protease Inhibitor Mixture (Sigma–Aldrich).

During the enrichment phase (round 1 only), ∼4 × 10^12^ phage, representing 100-fold coverage of the library, were negatively selected for 2 h at RT with a mixture of 500 μl of unconjugated washed Dynabeads, 500 μg of free streptavidin protein (RayBiotech, Norcross, GA), and 1 μg of heat-denatured HLA-A3 conjugated to Dynabeads to remove any phage recognizing either streptavidin or denatured HLA-A3. After negative selection, beads were isolated with a DynaMag-2 magnet (Life Technologies), and the supernatant containing unbound phage was transferred for positive selection for 1 h at RT against the 0.5 μg of mutant pHLA-A3 conjugated to Dynabeads. Prior to elution, beads were washed 10 times with 1 ml of TBST (1× TBS with 0.5% Tween 20). Phage were eluted by resuspending the beads in 1 ml of 0.2 m glycine, pH 2.2. After a 10-min incubation, the solution was neutralized by the addition of 150 μl of 1 m Tris, pH 9.0. Neutralized phages were used to infect 10-ml cultures of mid-log phage SS320s, with the addition of M13K07 helper phage (MOI of 4) and 2% glucose. Bacteria were then incubated as described previously, and the phages were precipitated the next morning with PEG/NaCl.

During the competitive phase (rounds 2–4), phage from the previous round was subjected to negative selection against heat-denatured HLA-A3, unrelated native pHLA-A3, and free streptavidin. After negative selection, beads were isolated with a DynaMag-2 magnet, and unbound phage was transferred for positive selection. This was performed by co-incubating phage simultaneously with 0.5 μg of mutant pHLA-A3 conjugated to the magnetic Dynabeads and 1 μg of WT pHLA-A3 conjugated to streptavidin-coated agarose beads. Prior to elution, beads were washed 10 times in 1 ml of TBST. Phage were eluted and used to infect mid-log phase SS320 cells as described above.

During the final selection phase (rounds 5 and 6), phage from the previous round was subjected to two sequential negative selection steps against 0.5 μg of WT pHLA-A3 each, followed by positive selection against 0.5 μg of mutant pHLA-A3.

### PCR and sequencing

Monoclonal phage supernatant (1 μl in a total reaction volume of 20 μl) was used as input into OneTaq® 2× Master Mix with Standard Buffer (New England Biolabs) with primers flanking the scFv in our phage library and the PCR run according to the manufacturer's protocol. PCR product was submitted to Genewiz (South Plainfield, NJ) for Sanger sequencing. Unique phage clones were determined using the online tool CD-HIT (65) and amplified in bacteria for further assays.

### ELISA

Wells of EvenCoat streptavidin-coated, 96-well plates (R&D Systems, Minneapolis, MN) were coated with 50 ng of biotinylated pHLA-A3 in 50 μl of blocking buffer (PBS with 0.5% BSA, 2 mm EDTA, and 0.1% sodium azide) overnight at 4 °C. Plates were briefly washed with 1× TBST (TBS + 0.05% Triton X-100). Phage were serially diluted to the specified concentrations in TBST, and 50 μl was added to each well. Phage were incubated for 2 h at RT, followed by six washes with TBST using a BioTek 405 plate washer (BioTek, Winooski, VT). The bound phage were incubated with 50 μl of rabbit anti-fd/M13 bacteriophage antibody (Novus Biologicals, Centennial, CO) diluted 1:3,000 in TBST for 1 h at RT, followed by washing an additional six times and incubation with 50 μl of goat anti-rabbit IgG horseradish peroxidase (Thermo Fisher Scientific) diluted 1:10,000 in TBST for 1 h at RT. After a final six washes with TBST, 50 μl of TMB substrate (BioLegend) was added to the well, and the reaction was quenched with an equal volume of 1 n sulfuric acid. Absorbance at 450 nm was measured with a Synergy H1 Multi-Mode Reader (BioTek).

Monoclonal phage ELISA was performed by selecting individual colonies of SS320 cells transformed with a limiting dilution of phage obtained from the final selection. Individual colonies were inoculated into 200 μl of 2xYT medium containing 100 μg/ml carbenicillin and 2% glucose and grown for 3 h at 37 °C. The cells were then infected with 1.6 × 10^7^ M13K07 helper phage and incubated for 1 h at 37 °C with shaking. The cells were pelleted, resuspended in 300 μl of 2xYT medium containing carbenicillin (100 μg/ml) and kanamycin (50 μg/ml), and grown overnight at 30 °C. Cells were pelleted, and the phage-laden supernatant was used for ELISA as described above.

Recombinant scFv ELISAs were performed similarly with serial dilution of scFvs in TBST with incubation in appropriate wells for 2 h at RT. After washing, protein L (Thermo Fisher Scientific) at 1 μg/ml in TBST was incubated in the wells for 1 h at RT. Following washing, anti-protein L–horseradish peroxidase (Abcam, Cambridge, UK) at 1:10,000 in TBST was added to the wells for 1 h at RT. ELISA was exposed using TMB and quenched with an equal volume of 1 n sulfuric acid.

### Peptide pulsing and flow cytometry

T2A3 cells, T2 cells were washed once with PBS and once with RPMI 1640 without serum before incubation at 1 × 10^6^ cells/ml in serum-free RPMI 1640 containing 50 μg/ml peptide and 10 μg/ml human β_2_-microglobulin (ProSpec, East Brunswick, NJ) overnight at 37 °C. The pulsed cells were pelleted, washed once in PBS, and resuspended in 100 μl of stain buffer (PBS containing 0.5% BSA, 2 mm EDTA, and 0.1% sodium azide). Phage staining was performed with 10 μl of phage (∼1 × 10^10^ phage) for 1 h on ice, followed by one wash with cold stain buffer. Cells were then stained with 1 μl of rabbit anti-M13 antibody (Novus Biologicals, Centennial, CO) on ice for 1 h, washed once, and stained with PE-conjugated anti-rabbit (BioLegend) on ice for 1 h, followed by the addition of LIVE/DEAD^TM^ Fixable Near-IR Dead Cell Stain (Thermo Fisher Scientific) for 10 min at RT. Cells were washed once in stain buffer before analysis.

ScFv staining was performed with 1 μg of scFv for 1 h on ice in 100 μl, followed by wash with stain buffer and then staining with 1 μl of PE-conjugated anti-DYKDDDDK (BioLegend) for 1 h on ice, followed by the addition of LIVE/DEAD dye. Cells were washed once in stain buffer before analysis. Other T2A3 staining was performed with a pan-HLA-A/B/C antibody W6/32 PE conjugate (BioLegend) and anti-HLA-A3 clone GAP.A3 PE conjugate (Thermo Fisher Scientific) followed by the addition of LIVE/DEAD dye. Stained T2A3 cells were analyzed using an LSRII flow cytometer (BD Biosciences).

### scFv expression and purification

The scFv sequences were cloned into pcDNA3.1 vector with an N-terminal IL2 secretion sequence and a C-terminal His tag (GenScript, Piscataway, NJ). Expression was done using a protocol adapted from Ref. 66. Briefly, 1 mg of purified plasmid DNA was transfected with polyethyleneimine at a ratio of 1:3 into 1 liter of Freestyle293 cells at a density of 2–2.5 × 10^6^ cells/ml and incubated at 37 °C for 7 days. The medium was harvested via centrifugation and filtered, and the scFv was purified via stepwise affinity and size-exclusion chromatography on a Superdex 75 Increase column (GE Healthcare). Alternatively, recombinant scFvs containing a C-terminal FLAG^TM^ tag (DYKDDDDK) were produced in *E. coli* by AxioMx (AxioMx, Abcam, Cambridge, UK).

### Surface plasmon resonance

All SPR experiments were performed on a Biacore T200 (GE Healthcare) optical biosensor at 25 °C. Approximately 135–145 response units of biotinylated mutant and WT pHLA-A3 were loaded onto Fc 2 and 4, respectively, of a streptavidin sensor chip. Single-cycle kinetics were performed with increasing concentrations of purified scFvs flowed over Fc 1–4. Binding responses for kinetic analysis were both reference-subtracted and blank-subtracted. The curves for E10 and cl. 7 were fit with a 1:1 binding model, whereas cl. 3 was fit with a heterogeneous ligand model using Biacore Insight evaluation software.

### Complement-dependent cytotoxicity assay

scFvs were conjugated to an anti-V5 mouse mAb clone SV5-Pk1 (Thermo Fisher Scientific) at a 2:1 molar ratio overnight at 4 °C. Conjugated scFvs or control anti-HLA antibody W6/32 (BioLegend) were serially diluted in serum-free RPMI 1640 on ice. Baby rabbit complement (Cedarlane Laboratories, Burlington, Canada), resuspended with ice-cold double-distilled H_2_O, was added to the serially diluted antibody conjugates before transferring 60 μl to a 96-well plate. An additional 40 μl of prechilled peptide-pulsed T2A3 cells containing 20,000 cells was transferred to the plate and mixed with gentle pipetting. In all cases, a final complement concentration of 10% was used for the assay. The plate was incubated at 37 °C for 1 h and subsequently read by the CellTiter-Glo® Luminescent Cell Viability Assay (Promega, Madison, WI) as per the manufacturer's instructions. Cell death was calculated by first normalizing each of the three cell subtypes (cells pulsed with different peptides or β_2_-microglobulin–only control) to the maximum luciferase signal (no antibody control), followed by subtraction from 100%. Specific cell death was defined as the cell death at a particular antibody concentration divided by the maximum cell death observed after treatment with W6/32 antibody for each of the given three cell subtypes.

### Affinity maturation

A library based on the E10 scFv sequence was synthesized by GeneArt Thermo Fisher with NNN degenerate codons in all six CDRs. The library was skewed toward the inclusion of the WT E10 nucleotide in each of the varied positions at a rate of 94–98%. Affinity maturation of the E10 scFv was performed by AxioMx (a subsidiary of Abcam) as follows. The E10 variant DNA library was cloned into a phagemid plasmid and used to generate an E10 variant phage display library. This library was subjected to three rounds of panning, each consisting of negative selection against 1 μg of WT pHLA-A3 followed by positive selection against the mutant pHLA-A3. The amount of mutant pHLA-A3 used for positive selection for each of the three rounds was 1 μg, 100 ng, and 10 ng, respectively. Following panning, individual phage clones were sequenced and tested via ELISA for binding to the mutant pHLA-A3 and WT pHLA-A3 monomers.

### Calculation of aggregation score

The aggregation score, AggScore, as defined by Sankar *et al.* (54) and implemented in the Schrödinger's Biologics Suite was used to calculate the aggregation propensity of the selected scFv. The method used the three-dimensional structure to estimate the distribution of hydrophobic and electrostatic patches on the surface of the protein.

### Modeling

All modeling was performed using the Biologics Suite, accessed using the Maestro (2018-3) interface (Schrödinger). Programs used for modeling were accessed through SBGrid (67). Antibody structure prediction for E10 was performed using BioLuminate (Schrödinger) (55). The framework selected was the crystal structure of 4D5 (PDB entry 1FVC) (68), given that our phage display library was based on this framework. BioLuminate uses a combination of structure clustering, sequence similarity, and geometry matching of the adjacent residues on either side of each loop to select CDR loops for grafting from the curated antibody database. A single model of the scFv was then used for docking to the mutant pHLA-A3 complex. Prior to docking, the mutant pHLA-A3 was prepared using the Protein Preparation Wizard, selecting the most exposed of the two conformations of the phenylalanine residue of interest (69). The predicted E10 structure was then docked to the prepared mutant pHLA-A3 complex using the antibody mode in Piper (56), which restricts the binding interface to the CDRs, and removing β-microglobulin as a potential docking surface. 20 poses were generated. Poses that did not contain direct interactions with the phenylalanine residue of the peptide were discarded. The four best poses were then minimized using Prime (Schrödinger). The poses were then evaluated for their ability to explain observed affinity differences in the affinity-matured clones. Overall and apolar buried surface area was calculated using Get Area (70). Phenylalanine buried surface area was calculated using PISA (71).

## Author contributions

M. S. M., J. D., M. S. H., A. D. S., N. P., K. W. K., B. V., S. Z., and S. B. G. conceptualization; M. S. M. and S. B. G. data curation; M. S. M., J. D., M. S. H., A. D. S., M. M., K. W. K., B. V., and S. Z. formal analysis; M. S. M., J. D., M. S. H., A. D. S., and M. M. validation; M. S. M., J. D., M. S. H., M. M., and S. B. G. investigation; M. S. M., J. D., and M. S. H. visualization; M. S. M., J. D., and M. S. H. methodology; M. S. M. and J. D. writing-original draft; M. S. H., A. D. S., K. W. K., B. V., S. Z., and S. B. G. writing-review and editing; K. W. K., B. V., S. Z., and S. B. G. supervision; K. W. K., B. V., and S. B. G. funding acquisition; S. Z. and S. B. G. project administration.

## Supplementary Material

Supporting Information

## Footnotes

4 The abbreviations used are:

APC: adenomatous polyposis coli
ADC: antibody-drug conjugates
BiTE: bispecific T-cell engager
CAR-Ts: chimeric antigen receptor T-cells
CDC: complement-dependent cytotoxicity
CDR: complementarity-determining region
cl.: clone
DF: desmoid type fibromatosis
HLA: human leukocyte antigen
MANA: mutation-associated neoantigens
MHC: major histocompatibility complex
pHLA: peptide-bound HLA complex
RMSD: root mean square deviation
scFv: single-chain variable fragment
SPR: surface plasmon resonance
TCR: T-cell receptor
WT1: Wilms tumor 1
TRIM: trinucleotide mutagenesis
Bicine: *N*,*N*-bis(2-hydroxyethyl)glycine
PDB: Protein Data Bank
RT: room temperature
MOI: multiplicity of infection
PE: phycoerythrin.
