# Supplementary material for: An engineered antibody fragment targeting mutant β-catenin via major histocompatibility complex I neoantigen presentation
Source: J Biol Chem. 2019 Nov 5;294(50):19322–34. doi: 10.1074/jbc.RA119.010251 (PMC6916501; doi:10.1074/jbc.RA119.010251)
Supplement: Supporting Information [file supp_294_50_19322__index.html]

An engineered antibody fragment targeting mutant β-catenin via Major Histocompatibility Complex I neoantigen presentation — Targeting mutant β-catenin — An engineered antibody fragment targeting mutant β-catenin via major histocompatibility complex I neoantigen presentation — Targeting mutant β-catenin — Supporting Information 

# An engineered antibody fragment targeting mutant β-catenin via major histocompatibility complex I neoantigen presentation

## Supporting Information

- Supporting Information (to be published online) - Supporting Information including sequences and additional figures
