# Supplementary material for: An engineered antibody fragment targeting mutant β-catenin via major histocompatibility complex I neoantigen presentation
Source: J Biol Chem. 2019 Nov 5;294(50):19322–34. doi: 10.1074/jbc.RA119.010251 (PMC6916501; doi:10.1074/jbc.RA119.010251)
Supplement: Supporting Information [file supp_RA119.010251_154397_2_supp_418434_pzxqvp.pdf]

Supporting Information

An engineered antibody fragment targeting mutant β-catenin via HLA neoantigen presentation

Michelle S. Miller[1‡], Jacqueline Douglass[2‡], Michael S. Hwang[2], Andrew D. Skora[2#], Michael Murphy[3], Nickolas Papadopoulos[2], Kenneth W. Kinzler[2], Bert Vogelstein[2,4], Shibin Zhou[2], Sandra B. Gabelli[1,5,6*]

From the [1]Department of Oncology, Johns Hopkins University School of Medicine, Baltimore, MD, 21287, USA; [2]Ludwig Center, Sidney Kimmel Comprehensive Cancer Center, Johns Hopkins University School of Medicine, Baltimore, MD, 21287, USA; [3]GE Healthcare Life Sciences, 100 Results Way, Marlborough, MA, 01752, USA; [4]Howard Hughes Medical Institute, Johns Hopkins University School of Medicine, Baltimore, MD, 21287, USA; [5]Department of Medicine, Johns Hopkins University School of Medicine, Baltimore, MD, 21287, USA; [6]Department of Biophysics and Biophysical Chemistry, Johns Hopkins University School of Medicine, Baltimore, MD, 21205, USA

Running title: *Targeting mutant β-catenin*

[#]Present address: Lilly Biotechnology Center, Eli Lilly and Co, San Diego, CA, 92121, USA
[‡]These authors contributed equally to this work.

*To whom correspondence should be addressed: Sandra B. Gabelli: Johns Hopkins University School of Medicine, Baltimore, MD, 21205, USA; gabelli@jhmi.edu

Fig. S1. 2mFo-DFc map of β-catenin S45F mutant peptide. A.

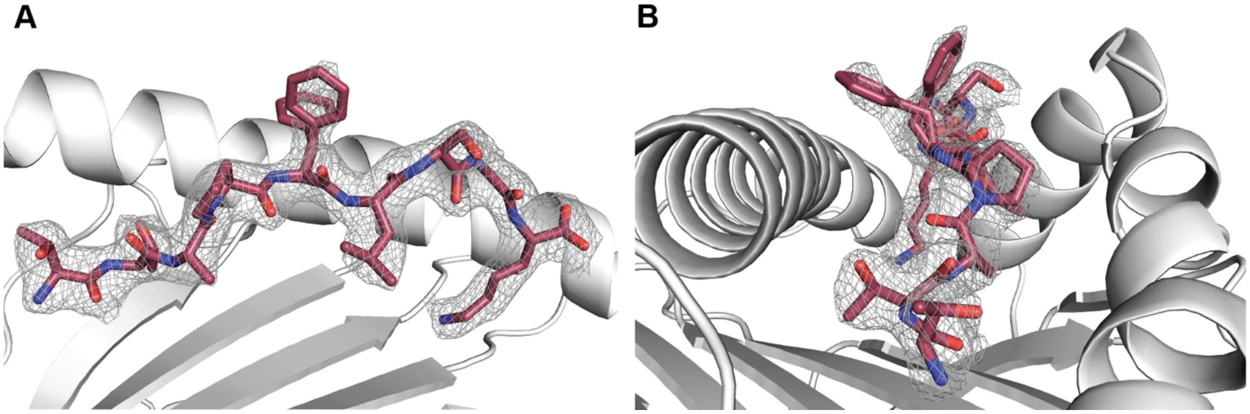

Fig. S2. Amino acid sequences of S45F mutant β-catenin$_{41\text{-}49}$pHLA-A*03:01 specific scFvs.

E10
DIQMTQSPSSLSASVGDRVTITCRASQDVNTAVAWYQQKPGKAPKLLIYSASFLYSGVPSRFSGSRSGTDFTL
TISSLQPEDFATYYCQQSYYSPPTFGQGTKVEIKRTGGGSGGGASEVQLVESGGGLVQPGGSLRLSCAASGFN
INNTYIHWVRQAPGKGLEWVASIYPTDGYTRYADSVKGRFTISADTSKNTAYLQMNSLRAEDTAVYYCSRTY
YSYYSAMDVWGQGTLVTVSS

cl. 3
DIQMTQSPSSLSASVGDRVTITCRASQDVNTAVAWYQQKPGKAPKLLIYSAYFLYSGVPSRFSGSRSGTDFTL
TISSLQPEDFATYYCQQIYTSPITFGQGTKVEIKRTGGGSGGGGSGGGASEVQLVESGGGLVQPGGSLRLSCA
ASGFNFITTGMHWVRQAPGKGLEWVARIGPGSDYTNYADSVKGRFTISADTSKNTAYLQMNSLRAEDTAV
YYCSRYYYASALDYWGQGTLVTVSS

cl. 4
DIQMTQSPSSLSASVGDRVTITCRASQDVNTAVAWYQQKPGKAPKLLIYSASFLYSGVPSRFSGSRSGTDFTL
TISSLQPEDFATYYCQQRAYFPITFGQGTKVEIKRTGGGSGGGGSGGGASEVQLVESGGGLVQPGGSLRLSC
AASGFNFSDYGMHWVRQAPGKGLEWVAMLIPASGYTNYADSVKGRFTISADTSKNTAYLQMNSLRAEDTA
VYYCSRGWSYYMDYWGQGTLVTVSS

cl. 7
DIQMTQSPSSLSASVGDRVTITCRASQDVNTAVAWYQQKPGKAPKLLIYSASFLYSGVPSRFSGSRSGTDFTL
TISSLQPEDFATYYCQQQYAYTPITFGQGTKVEIKRTGGGSGGGGSGGGASEVQLVESGGGLVQPGGSLRLS
CAASGFNVWSYGIHWVRQAPGKGLEWVAGVTPDGSYTYYADSVKGRFTISADTSKNTAYLQMNSLRAEDT
AVYYCSRSYGWAMDYWGQGTLVTVSS

cl. 9
DIQMTQSPSSLSASVGDRVTITCRASQDVNTAVAWYQQKPGKAPKLLIYSAYFLYSGVPSRFSGSRSGTDFTL
TISSLQPEDFATYYCQQIHYKPLTFGQGTKVEIKRTGGGSGGGGSGGGASEVQLVESGGGLVQPGGSLRLSCA
ASGFNVAWYSIHWVRQAPGKGLEWVAQVYGGSSYTYYADSVKGRFTISADTSKNTAYLQMNSLRAEDTAV
YYCSRDFYSSGMDYWGQGTLVTVSS

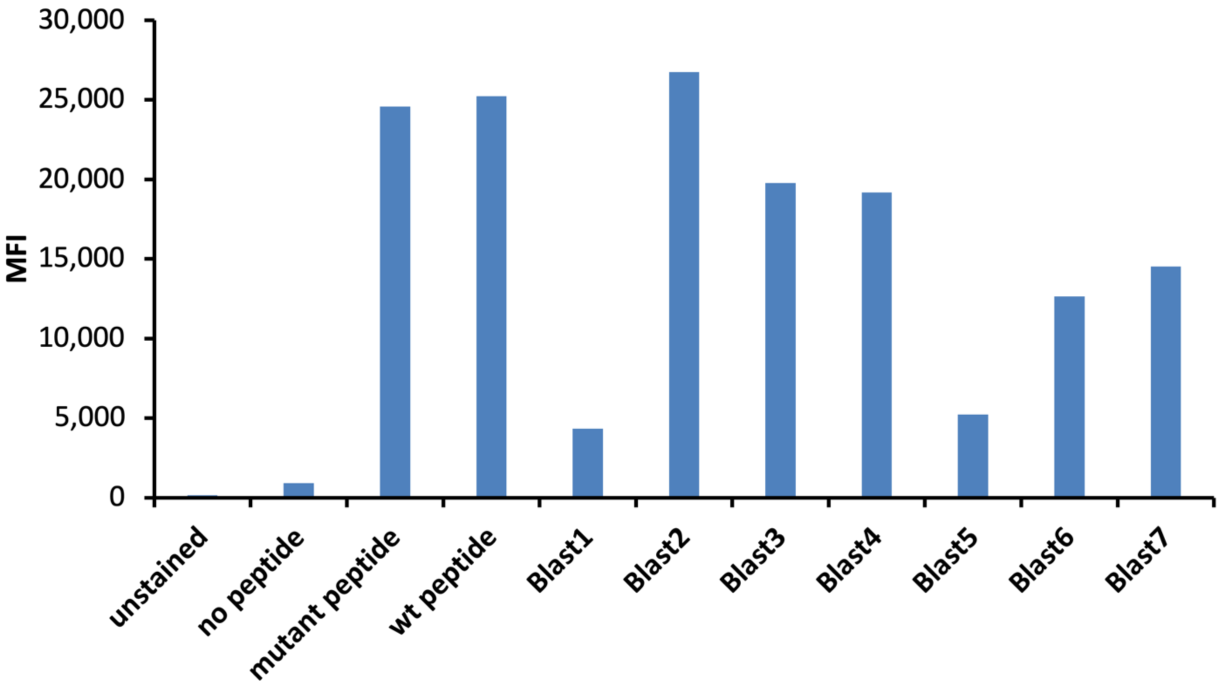

Fig. S3. Anti-HLA-A3 staining to assess peptide pulsing of CTNNB peptides and BLAST peptides. T2A3 cells were peptide-pulsed overnight at 37°C in serum-free media with either the CTNNB mutant S45F peptide (TTAPFLSGK) and β2M, CTNNB wildtype peptide (TTAPSLSGK) and β2M, or β2M only. Cells were incubated with anti-HLA-A3 antibody clone GAP.A3 PE conjugate. Cells were stained with a live/dead Near-IR dye, washed, and analyzed by an LSRII flow cytometer.

Table S1. Blast Peptides. BLAST peptides were identified by a BLASTp search of the CTNNB S45F mutant peptide (TTAPFLSGK) against the normal human proteome. Hits were then analyzed by netMHCv4.0 for binding to HLA-A*03:01.

Peptide Name	Peptide	Predicted Affinity (nM)	Protein
Blast1	QLLDFLSGK	91.7	PCNA-interacting partner isoform 1 [Homo sapiens]
Blast2	SLNPKFLSGK	16.9	ephrin-B1 precursor [Homo sapiens]
Blast3	IIYNFLSGK	7.3	orexin receptor type 2 isoform X1 [Homo sapiens]
Blast4	RTVTFLSGK	13.4	nuclear receptor subfamily 1, group D, member 2 [Homo sapiens]
Blast5	TAFDPFLGGK	178.5	heat shock protein 105 kDa isoform 3 [Homo sapiens]
Blast6	RIIPFLPGK	18.0	SH3 and PX domain-containing protein 2A isoform 1 [Homo sapiens]
Blast7	IQNPFLSSK	60.4	SCAN domain-containing protein 3 isoform 1 [Homo sapiens]
